# Noninvasive Electrical Stimulation Neuromodulation and Digital Brain Technology: A Review

**DOI:** 10.3390/biomedicines11061513

**Published:** 2023-05-23

**Authors:** Shuang Zhang, Yuping Qin, Jiujiang Wang, Yuanyu Yu, Lin Wu, Tao Zhang

**Affiliations:** 1 The School of Artificial Intelligence, Neijiang Normal University, Neijiang 641000, China; 2 The School of Life Science and Technology, University of Electronic Science and Technology of China, Chengdu 610056, China; 3 The NJNU-OMNISKY Smart Medical Engineering Applications Joint Laboratory, Neijiang Normal University, Neijiang 641004, China; 4 The High Field Magnetic Resonance Brain Imaging Laboratory of Sichuan, Chengdu 610056, China; 5 The Sichuan Institute for Brain Science and Brain-Inspired Intelligence, Chengdu 610056, China

**Keywords:** noninvasive, electrical stimulation, neuromodulation, digital brain, time domain, spatial domain, digital projection

## Abstract

We review the research progress on noninvasive neural regulatory systems through system design and theoretical guidance. We provide an overview of the development history of noninvasive neuromodulation technology, focusing on system design. We also discuss typical cases of neuromodulation that use modern noninvasive electrical stimulation and the main limitations associated with this technology. In addition, we propose a closed-loop system design solution of the “time domain”, “space domain”, and “multi-electrode combination”. For theoretical guidance, this paper provides an overview of the “digital brain” development process used for noninvasive electrical-stimulation-targeted modeling and the development of “digital human” programs in various countries. We also summarize the core problems of the existing “digital brain” used for noninvasive electrical-stimulation-targeted modeling according to the existing achievements and propose segmenting the tissue. For this, the tissue parameters of a multimodal image obtained from a fresh cadaver were considered as an index. The digital projection of the multimodal image of the brain of a living individual was implemented, following which the segmented tissues could be reconstructed to obtain a “digital twin brain” model with personalized tissue structure differences. The “closed-loop system” and “personalized digital twin brain” not only enable the noninvasive electrical stimulation of neuromodulation to achieve the visualization of the results and adaptive regulation of the stimulation parameters but also enable the system to have individual differences and more accurate stimulation.

## 1. Introduction

There is an increasing demand for quality of life and self-health improvement with the progression of society and the development of science. However, with the rapid development of society, the pressure is also increasing on people’s lives and survival, leading to functional neurological diseases, such as insomnia, chronic pain, and depression. Functional neuropathy has become a significant disease that endangers the quality of life and self-health, and it can lead to neurological diseases such as insomnia, chronic pain, Parkinson’s disease, and epilepsy, as well as mental health disorders such as schizophrenia, depression, and mania. These diseases also severely impact people’s overall quality of life and health. Neurological diseases are characterized by dysfunction in the nervous system, whereas mental health disorders mainly encompass cognitive, emotional, willpower, and behavioral disorders (clinical manifestations) caused by brain dysfunction. These diseases often lack a clear pathogenic focus and only manifest as neurological dysfunctions [1], making treatment difficult. Simultaneously, a large number of patients has these diseases. For example, the global incidence of Parkinson’s disease is two percent [2]. Patients over the age of 60 account for one percent of the global incidence, and people over 65 years old account for five percent. According to the 2021 China Seventh National Population Census data, the population aged 60 and above is 264.02 million, accounting for 18.7% of the total population. Among them, 190.64 million people were aged 65 and above, accounting for 13.5% of the total population [3,4]. China now represents an aging population. Further, the incidence of epilepsy is 0.88% of the total population [5], which is more than ten million people in China. Therefore, functional neurological diseases have become a principal concern in modern medicine.

Treating neurological diseases mainly depends on drug and surgical treatment to alleviate symptoms. However, taking antipsychotics for a long time makes the body easily resistant to drugs, which causes difficulties for patients later on in treatment and causes substantial psychological and physical harm to them [6,7]. Therefore, surgical resection is often used to deal with drug-resistant diseases [8]. Surgeons remove the focus tissues and organs through clinical surgery to achieve the control and treatment of patients’ diseases [9,10,11]. Moreover, surgical treatment easily damages other tissues and organs and is irreversible. Therefore, high precision is required for medical diagnosis and surgical procedures. Achieving this precision can pose difficulties because of the size and site of the resected tissue, other tissue injuries, a doctor’s experience, and so forth [8,12,13]. Simultaneously, surgery also brings patients great physical and psychological pain [14,15,16]. Thus, the medical community and researchers have been attempting to find a therapeutic schedule to reduce patients’ psychological and physical injuries with minor side effects.

The emergence of neuromodulation technology has significantly improved and promoted the maturity and development of modern functional neurosurgery, making functional neurosurgery one of the fastest-growing disciplines in the field of medical technology [17,18,19,20,21,22,23,24]. Neuromodulation technology allows drugs or physical signals to act directly on the target area in an invasive or noninvasive way, reducing other organ damage caused by drug overdose compared with oral drugs [8]. This technology, compared with surgery, lowers the difficulty of operation, avoids injuring other tissues by mistake, and relieves patients’ pain. Most importantly, the technology is reversible [17,22,23,24]. The goal of modern neuromedicine has become to seek a neural regulation mode with less injury to human tissues, high stimulation accuracy, simple operation, and no side effects, with the development of medical technology [25]. Noninvasive nerve regulation technologies, such as TES (tDCS and tACS) [26,27] and transcranial magnetic stimulation (TMS) [28], are widely used in clinical treatment. However, because tDCS will not make neurons produce action potential, and the current intensity is weak, the effect of tDCS in clinical treatment is less effective than TMS. However, as the tACS signal characteristics are changed, the stimulation depth of the system also deepens, and the regulatory targeting gradually affects the regulation of the neuronal function. In addition, TMS is prone to inducing organic changes in brain tissues; therefore, tACS is gaining popularity amongst clinicians.

This paper reviews the progress in the research on noninvasive deep brain stimulation neural regulation technology. Especially in the context of system design and precise targeting, it points out the advantages and disadvantages of existing paradigms. It combines the development trends of brain–computer interfaces to propose a closed-loop neural regulation system and personalized digital-twin brain architecture, providing a new direction for precise neural regulation.

**Figure A1:**
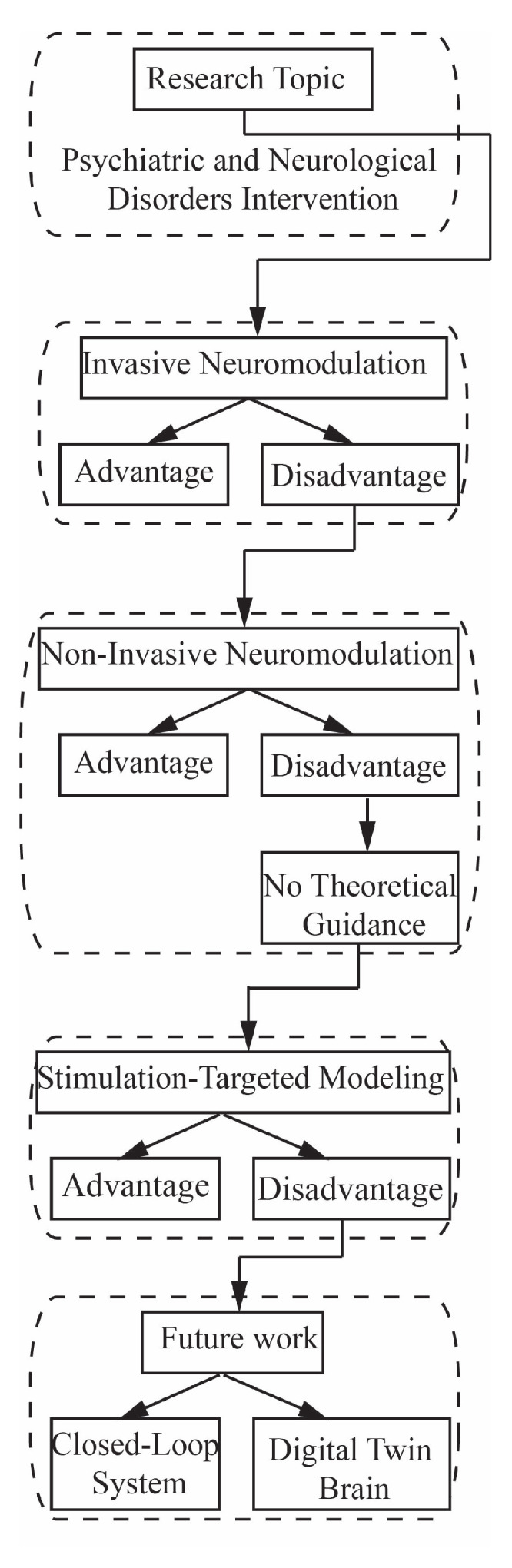
Literature analysis flowchart.

In this paper, we review the development and treatment needs of neurological and mental health disorders and summarize the problems and limitations of conventional treatment methods and invasive neural regulation techniques. From the perspective of minimally invasive properties and easy operation, this paper analyzes the development of a noninvasive neural regulation technique while acknowledging its limitations and drawing forth a review and analysis of digital human research. Furthermore, we propose future research directions based on the progress of noninvasive treatment and digital human research. The paper consists of eight sections: Section 1 introduces the market demand for neural regulation technology. Section 2 summarizes three invasive neuromodulation techniques. Section 3 summarizes the development of noninvasive neural regulation technology and its modern medical application. Section 4 discusses the existing problems and limitations in noninvasive neural regulation technology, including the bottleneck in theoretical research and development. Section 5 summarizes the development of noninvasive theory, specifically “digital human” technology. In Section 6, we propose research ideas for future work, including the design architecture of a closed-loop neural regulation system and a digital twin brain that combines structure and function. Section VII presents a series of discussions on the relevant research in this paper. The last section concludes the main points of this study (literature analysis flowchart shown in Figure A1).

## 2. Invasive Neuromodulation Method

Presently, the commonly used neuromodulation technologies in clinical medicine mainly include three significant directions: implantable drug delivery micropump neuromodulation [29,30,31], implantable neural electrical stimulation technology [17,18,19,20,21,22], and deep brain stimulation [17,18,19,20,21,22,23,24,25,29,30,31,32,33,34].

### 2.1. Implantable Drug-Delivery Micropump Neuromodulation

Implantable drug-delivery micropump neuromodulation refers to the implantation of the drug-delivery micropump regulation system into the subarachnoid space for continuous drug administration to the nervous system [29,30,31]. Other physical methods have been tested to replace the drug-delivery micropump regulation system because it will make patients resistant to drugs, leading to the inefficacy of the drugs and the need to replace it with another treatment.

### 2.2. Implantable Neural Electrical Stimulation Technology

Neural electrical stimulation technology regulates diseases by implanting a pulse microcurrent generator into the human body to continuously stimulate the brain, spinal cord, peripheral nerves, nerve plexus, and autonomic nerves [17,18,19,20,21,22]. For example, in Blade volume sensors [35] and cochlear implants [36,37], external electrical signals are used to improve or repair the function of certain organs in patients.

### 2.3. Deep Brain Stimulation

Deep brain stimulation (DBS) is a new way to intervene and treat brain diseases by implanting stimulation electrodes in the brain through stereotactic positioning. Compared with conventional surgical treatment, DBS has the advantages of reversibility, adjustability, and minimal invasiveness [17,18]. The DBS system consists of stimulation electrodes implanted into specific deep brain areas, pulse generators implanted subcutaneously in the clavicle area, and external programmable controllers. DBS releases chronic microcurrent to a specific area deep in the brain by the stimulating electrode during the implementation of stimulation, thus achieving the treatment of diseases in this area [17]. DBS has been approved by the US FDA for the clinical adjuvant treatment of patients with Parkinson’s disease and severe depression [32] and is expected to become a new approach for treating drug-resistant epilepsy [18,33]. However, as the implantable neuromodulation technology causes postoperative sequelae in patients and because of the limitation of the battery capacity of the implantable device, performing multiple operations is often necessary to replace the battery during a treatment period. This limitation increases the economic burden on patients and makes patients fear the surgery. Therefore, noninvasive transcranial deep brain electrical stimulation technology has become an important developmental direction of current neuromodulation technology [34].

## 3. Research Progress on Noninvasive Electrical Stimulation Neuromodulation

### 3.1. Application of Modern Medical Electrical Stimulation Technology

The earliest noninvasive electrical stimulation technology can be traced back to 46 AD, when Scibonius Largus, a pharmacist from Neron and doctor of the Roman Emperor Claudius, used the current released by electric eels to treat headaches and gout [38]. This was the first recorded application of noninvasive neural regulation for the treatment of functional neurological disorders. In 1777, Cavallo et al., published a study report on the application of electrical stimulation for the treatment of a variety of complex diseases, which detailed electrical stimulation techniques applied for the treatment of paralysis, epilepsy, deafness, chorea, blindness, glandular enlargement, and rheumatism. He was the first to recommend using electricity for artificial respiration, further expanding the clinical applications of electrical stimulation [39]. In 1818, Andrew Ure selected a prisoner’s corpse shortly after hanging and used direct current to stimulate its chest; it was found that the muscles of the corpse contracted upon stimulation, which indicated the resuscitation effect of electrical stimulation on the heart of patients without damaged organs, providing the earliest evidence for electrocardiac resuscitation [40]. In 1870, Fritsch and Hitzig found that a dog’s legs twitched during stimulation when a current was used to stimulate the motor cortex [41]. For the first time, it was proven that electrical stimulation could affect the motor nerve response of animals. In 1874, Roberts Bartholow carried out an electrical stimulation experiment on living humans. During the experiment, the unclear signal control induced a grand epileptic seizure [42], demonstrating that electrical stimulation was effective in regulating human neural functions; it also showed that inaccurate stimulation was more likely to induce new nervous system disease. In 1963, Jacobsen et al., conducted a study on the treatment of mental health disorders using electrical stimulation. By analyzing the effects before and after stimulation, it was found that stimulation had a significant effect on the treatment of mental health disorders [43]. This report was the first to treat mental health diseases using electrical stimulation.

### 3.2. Application of Electrical Stimulation Technology in Modern Medicine

**Figure 1:**
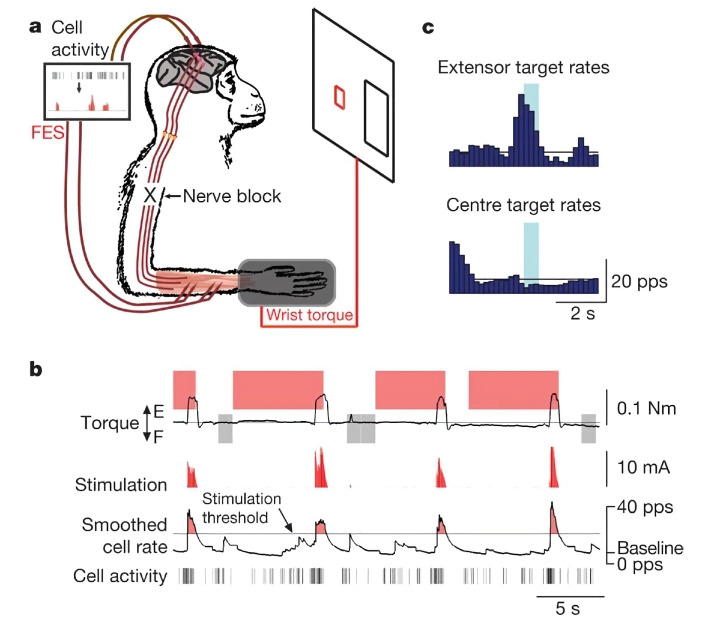
**Electric stimulation improves paralysis caused by spinal cord injury.** (**a**). The activity of the cerebral cortex cells is transformed into functional electrical stimulation (FES). (**b**). An electrical stimulation signal used to control wrist paralysis. (**c**). Statistical histogram for feeding back the effectiveness of functional electrical stimulation. Reprinted (adapted) with permission from Ref. [23]. Copyright ©2020, Springer Nature.

**Figure 2:**
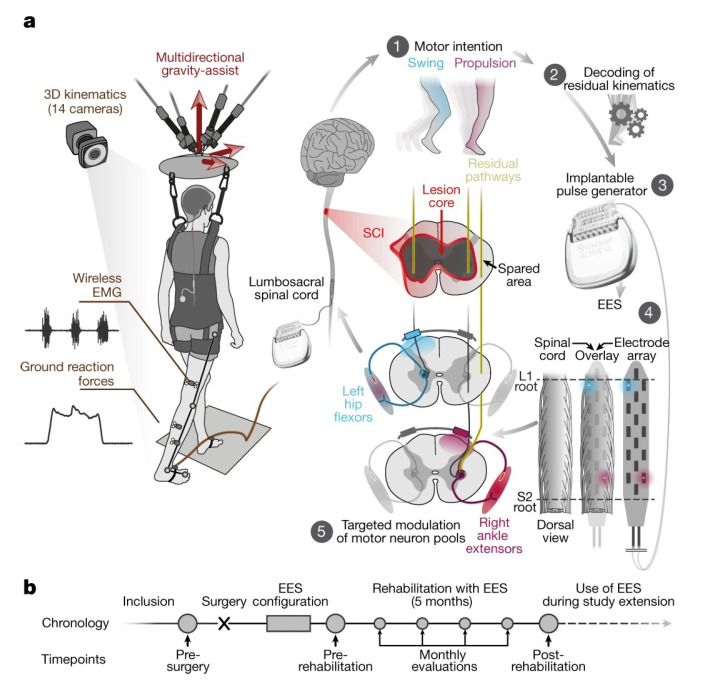
**Design scheme of electric stimulation exercise recovery.** (**a**). Targeted nerve stimulation in patients with spinal cord injury can return walking ability. During ground-assisted movement, the trunk’s multidirectional aids help maintain body movement navigation from multiple directions. Real-time recording of 3D motion status, ground reaction force, and electromyography evaluate the effect of post-stimulation movement recovery. (**b**). Study timeline. Reprinted (adapted) with permission from Ref. [44]. Copyright ©2022, Springer Nature.

In 2008, Chet T. Moritz of the University of Washington, while studying the effect of electrical stimulation on spinal cord injury by applying electrical stimulation therapy to a monkey paralyzed by spinal cord injury, found that monkeys were able to control computer cursors and robotic arms based on stimulation signals [23] (Figure 1). This demonstrated the positive role of electrical stimulation in helping repair functional damage to the nervous system. In 2018, a study in Nature reported that three patients with spinal cord injury gradually regained the ability to walk with assistive devices after receiving targeted spinal cord electrical stimulation (Figure 2). This result demonstrated that the use of electrical stimulation after spinal cord injury also contributed to human functional recovery and provided a technical framework for the later treatment and rehabilitation of spinal cord injury patients [44]. In the same year, they also published in Nature Neuroscience that the previous stimulation programs for walking recovery were less effective because they interfered with the patient’s perception of limb position. The short stimulation only promoted movement and retained the sensory signals from the legs [45]. Therefore, this result shows that the feedback of stimulation precision on the stimulation effect is essential.

The focusing accuracy of electrical stimulation signals has become a significant obstacle in its use for targeting the brain because the human skull has a strong shielding effect for electric currents, and brain tissue has strong diffusion and reuse of currents [46,47]. These characteristics limit the ability to precisely regulate electrical stimulation intended for this purpose.

**Figure 3:**
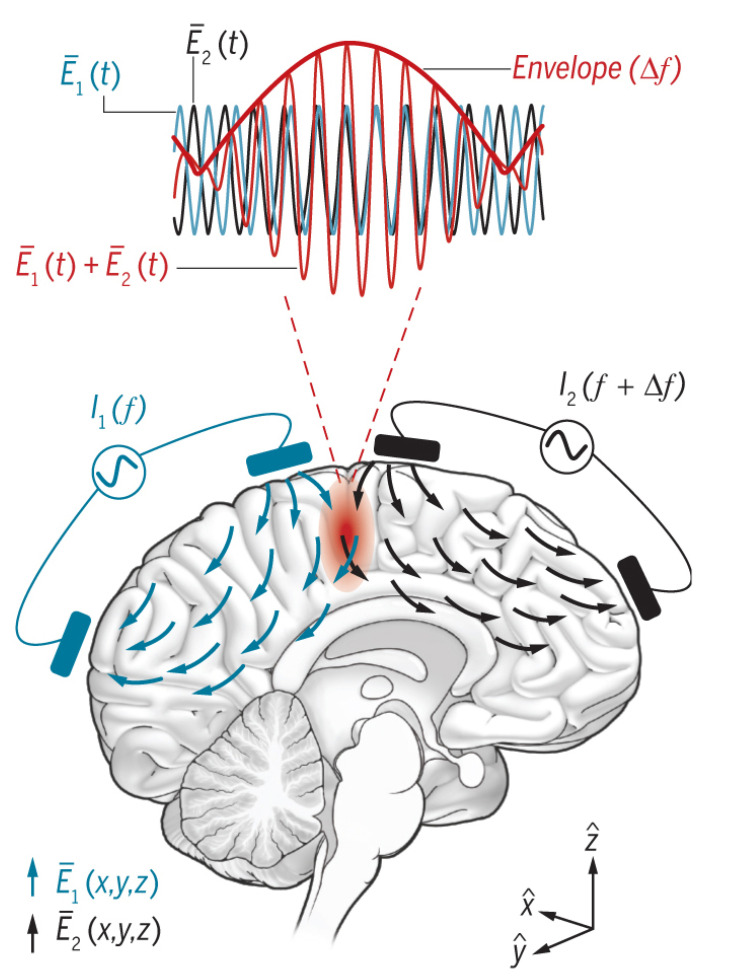
**Noninvasive deep brain stimulation.** Alternating currents signals of $I_{1}$ and $I_{2}$ are injected into electric field vectors $\bar{E_{1}}\left(x,y,x\right)$ and $\bar{E_{2}}\left(x,y,x\right)$ with frequencies of $f_{1}$ and $f_{2}$, respectively, where $f_{2}=f_{1}+$ Δ*f*. A signal envelope is formed by utilizing the frequency difference between the two signals in the targeting area to achieve targeted stimulation. Reprinted (adapted) with permission from Ref. [24]. Copyright ©2021, Cell Press.

In 2017, researchers from the Massachusetts Institute of Technology and the Imperial College of Technology in the United Kingdom developed a new method to stimulate the areas under the cerebral cortex without surgery (Figure 3) [24]. The researchers placed two electrodes at a special position on the top of a small mouse head. In one experiment, the electrode on the skull side generated a current of 2010 Hz, while the opposite electrode excited a current of 2000 Hz using a high-frequency current with a slight difference. The researchers used this high frequency because it does not affect the neurons in the brain region they flow through. However, mutual interference occurs in the brain regions where these currents overlap. Neurons can sense the difference between the two currents with different frequencies and are affected by the 10 Hz current.

Furthermore, this method does not interfere with the tissues around the brain target. Additionally, the authors used a lentivirus gene vector with a high-speed optical switch to transfect a natural seaweed protein ChR2 (channelrhodopsin-2) into neurons using photogenetic technology to observe the stimulation effect. They achieved excitation–inhibition control of the action potential and synaptic conduction through current stimulation. Moreover, the strong signal attenuation caused by the skull resulted in the stimulation action area of the surface electrode mostly concentrated in the cerebral cortex [25,48,49,50]. Thus, the individual is more sensitive to functional neurological diseases induced by the cortex [51,52,53,54,55,56]. This technology can help millions of patients with brain diseases. However, the DBS technology is mostly used for diseases with lesion areas in the deep brain [51,52,53,57,58,59,60,61]. If the electrical stimulation of deep brain regions were to be achieved through surface stimulation outside the brain, many limitations of implantable equipment and the side effects and economic pressures of surgery would be avoided. This development could prove very beneficial for treating long-term mental health diseases.

**Figure 4:**
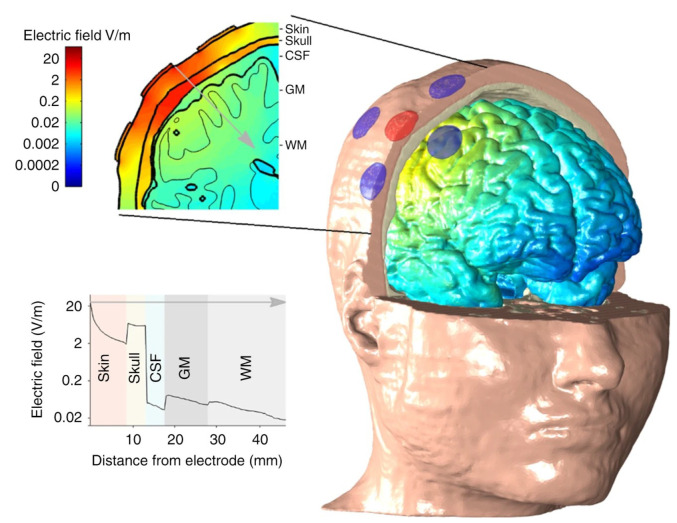
**Common cathode multi-electrode combined stimulation.**The figure shows the distribution of current signals in different brain tissues. Reprinted (adapted) with permission from Ref. [65]. Copyright ©2020, Springer Nature.

**Figure 5:**
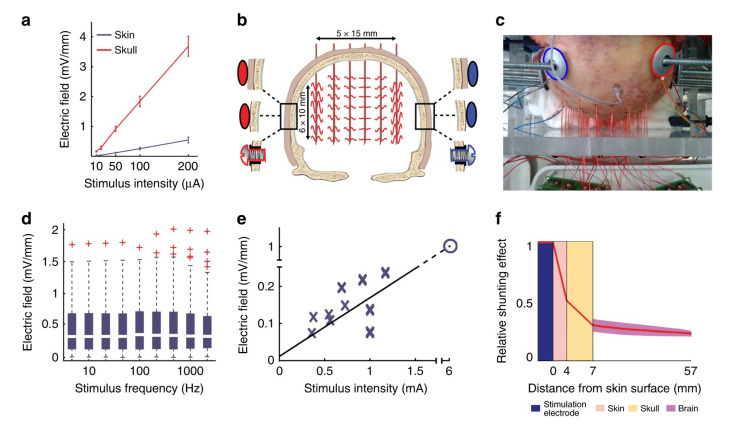
**Combined electric stimulation of free electrodes.** (**a**). Comparison of subcutaneous and percutaneous stimulation performances. (**b**). Schematic diagram of subcutaneous and percutaneous stimulation design for the head of a corpse. (**c**). A realistic picture of the corpse experiment. (**d**). Relationship between the voltage gradient inside the brain and the frequency of external stimulation. (**e**). Measured intracranial electric field intensity. (**f**). Decay of electric charge in brain tissues. Reprinted (adapted) with permission from Ref. [70]. Copyright ©2021, Springer Nature.

**Figure 6:**
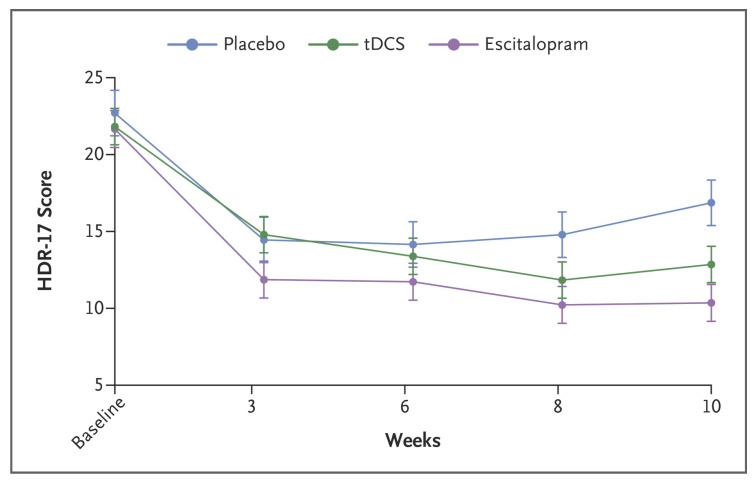
**Comparison of the improvement effects of conventional drugs and electrical stimulation on functional neurological diseases.** The figure shows almost no significant statistical difference in the inhibitory effect of tDCS and escitalopram on depressive disorder in short-term treatment (<6 weeks). Although the electrical stimulation technology is not more effective in treating depression than escitalopram in long-term treatment, the effect of tDCS is significantly better than placebo. Reprinted (adapted) with permission from Ref. [71]. Copyright ©2022, Massachussetts Medical Society.

Grossman et al., (2017), in their study, observed that researchers had used the combination of electrode groups and EEG multimodal stimulation to greatly improve the stimulation accuracy [62,63,64,65,66,67,68,69,70] (by about 2–3 cm^2^) and the stimulation depth (by about 3–4 cm) (Figure 4 and Figure 5). Electric stimulation technology, compared to the drug treatment, has similar effects on some functional neurological diseases such as depression and epilepsy. Nonetheless, this technology has fewer side effects and advantages, such as better pain reduction. Therefore, electric stimulation technology appears to have an overall better effect on treating functional neurological diseases (Figure 6) [71,72].

Clinical research shows that noninvasive neural regulation technology has advantages in regulating the cerebral cortex targeted activity (focal epilepsy) [73], regulating the hypodermic network activity (Parkinson’s disease) [74] with functional loss, improving adaptive balance (stroke and Alzheimer’s disease) [75] in the disconnected brain network, and inhibiting plastic change (pain and brain injury) [76].

Cortical electrical stimulation technology using surface electrodes for stimulation is mainly used to treat cortical-related diseases, including chronic pain [55], depression [54,61], epilepsy [51,53], and dyskinesia [56]. This technology has advantages in the application of noninvasive high-precision neuromodulation, such as simple operations, the possible change of the stimulation target point at will, and no surgery required, which avoids brain tissue damage and reduces complications [17,23,24]. Simultaneously, the high penetrability and signal controllability of AC stimulation [24] can be achieved by performing electric stimulation on deep brain areas using surface electrodes (The performance analysis of all the neuromodulation methods is shown in Table A1).

## 4. Problems with Noninvasive Electrical Stimulation Technology (Limitations)

Although electrical stimulation-based neuromodulation technology has shown great potential for treating functional neurological diseases, there are still many problems to be solved, mainly including the following:

1. Theoretical models are lacking, and the accuracy needs further improvement. Although the accuracy and depth of electrical stimulation have been improved, there is no clear theoretical model, and the mechanism of electrical signal transmission in the brain remains unclear. In particular, the envelope formed by multiple signals in the brain lacks accurate theoretical support for regulating target areas. Additionally, the accuracy of stimulation needs to be further improved. In particular, the human brain skull has high shielding, and the signal attenuation is large, while the deep brain region needs a higher depth of stimulation.
2. There is a scarcity of personalized brain stimulation maps. Currently, in the clinical localization technology of electrical stimulation, the conventional forehead localization method and the clinical trial method are also used for clinical stimulation. This method inevitably results in questions about the positioning accuracy of this technology. Notably, the pathogenesis and sites of different brain diseases are different. Moreover, there has been very little research on the mapping of personalized brain stimulation for different diseases. Accordingly, electrical stimulation also lacks a personalized scheme for different diseases.
3. The established model lacks accurate verification methods. The verification of head modeling has always been the focus of the scientific and medical communities. However, today, because of the limitations of ethics and medical technology, the verification of head modeling is difficult.

## 5. Research Progress in Noninvasive Electrical-Stimulation-Targeted Modeling

**Figure 7:**
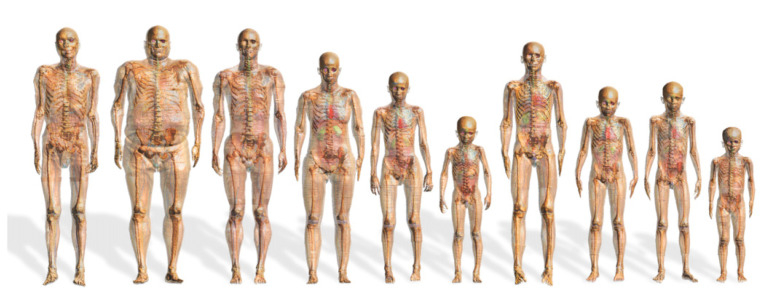
**Digital humans of different ages, shapes, and sex.** Images representing digital human model groups of different ages, sex and body types. Reprinted (adapted) with permission from Ref. [88]. Copyright ©2018, IOP Publishing Ltd.

The finite element modeling method is the most direct and effective method to provide an accurate simulation scheme for electrical stimulation. Neuromodulation modeling and source localization modeling are now almost complete by using a single-layer human head structure [77] and a multilayer sphere model [78,79] to reduce the difficulty of modeling and improve the computer computing speed of the r. The calculation results often have no practical significance because of the large difference between the single-layer human head structure model and the actual internal human brain structure [77]. Although the multilayer sphere model approximately represents the simple structural composition of the head, the model cannot effectively represent the signal transmission effects caused by the irregular structure of the head and different tissue distributions. Through verification, in simple localization simulation, the model error is greater than 1 cm, and the practical significance of the model is not great [78,79]. An attempt to build a real human brain simulation model through a digital human model has been conducted, considering the problems with a simplified model dataset [78,79,80,81,82,83,84,85,86,87,88,89,90,91,92,93,94,95,96,97,98,99,100,101,102,103,104,105,106,107,108,109,110,111,112,113,114,115] (Figure 7).

In building a real human brain simulation model, obtaining a complete and high-precision human data set is necessary, especially the complete data set of the human head. However, effective data segmentation must be conducted, and exported data sources can be used to implement secondary development. The digital human plan of Switzerland IT’IS Found [88,89] provides a complete data set of the human body and the organ segmentation scheme; however, these data can only be used under its self-developed Sim4Life platform. The data cannot be exported, limiting secondary research and development potential. Moreover, the software application is mainly centralized on MRI coil design and electromagnetic simulation; these procedures cannot be used to achieve electrical stimulation simulation design. Japanese NII [93], Korean ETRI [94,95,96,97], British NRPB [98], German Munchen, CST AG [98,100,101], American Penn State [99], U Texas Austin, and others [82,83,84,85,102,103,104,105,106,107,108,109,110,111,112,113,114] have also developed a digital human model. However, because of incomplete public data and a rough data segmentation scheme, numerous singularities are generated after data segmentation; therefore, it is difficult to achieve the reverse modeling of human organs. Even if reverse tissue modeling is achieved by simplifying the tissue structure, many head tissues and organs must be abandoned. The modeling accuracy is greatly reduced, which introduces certain difficulties in establishing the precisely targeted neuromodulation model.

**Figure 8:**
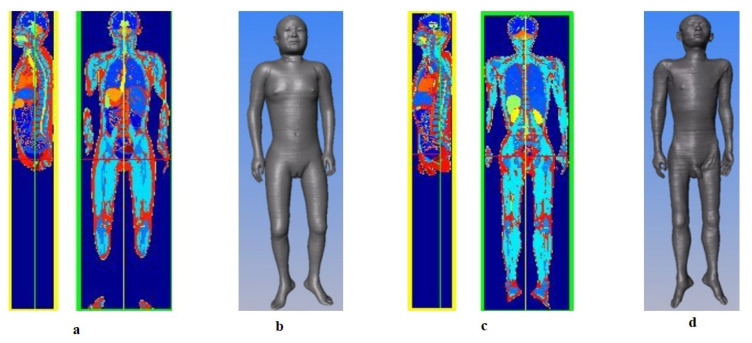
**Chinese digital human.** (**a**). Slice images of the female model. (**b**). Surface view of the female model created using Amira software. (**c**). Slice images of the male model. (**d**). Surface view of the male model created using Amira software. Reprinted (adapted) with permission from Ref. [90]. Copyright ©2021, IOP Publishing Ltd.

The Chinese digital human model (Figure 8) [87,90,91,92,115], developed under the leadership of Zhang Shaoxiang of the Army Military Medical University (formerly the Third Military Medical University), has high section accuracy and complete data. Above all, the image gray value segmentation method can be used for extraction from nearly 110 tissues/organs in the whole body and from 32 tissues/regions in the head, including some nerves, providing a data-based foundation for establishing a high-precision electrical stimulation neuromodulation model of the head.

**Figure 9:**
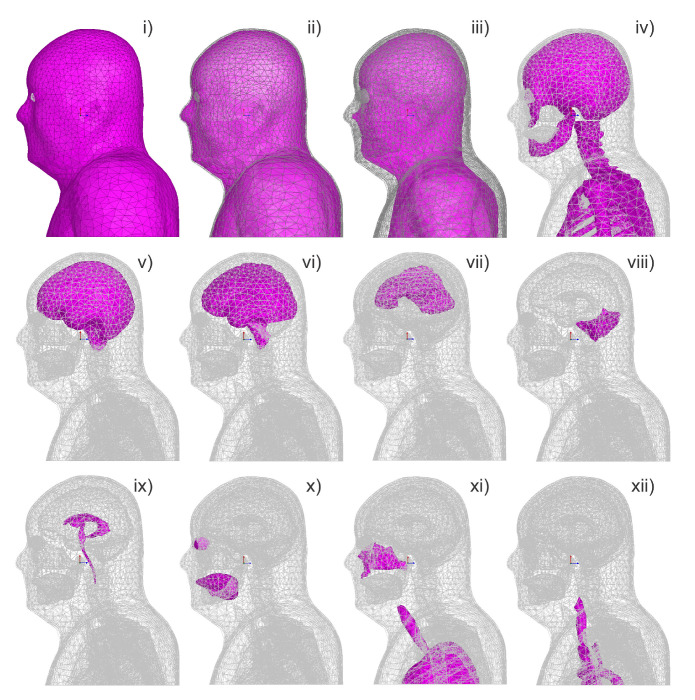
**Establishment of an electrical stimulation head model based on a digital human model.** A human head model used for tDCS calculation, in which each tissue is relatively independent and can be individually given material parameters, including: (**i**) skin; (**ii**) fat; (**iii**) muscle; (**iv**) bones; (**v**) CSF; (**vi**) cerebral cortex (gray matter); (**vii**) white matter; (**viii**) cerebellum; (**ix**) ventricles system; (**x**) eyes and tongue (separate tissues); (**xi**) sinus cavity, lungs, and trachea; (**xii**) aorta and cava superior. Reprinted (adapted) with permission from Ref. [85]. Copyright ©2022, Institute of Electrical and Electronics Engineers Inc.

**Figure 10:**
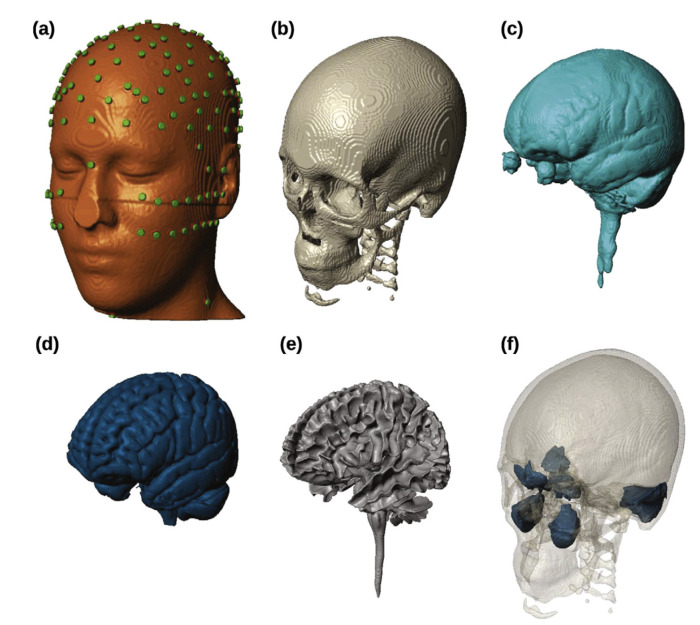
**Digital brain model based on brain VISA software (brainVISA 5.0.4, CEA, France)**. (**a**–**f**): scalp (with 231 electrodes placed), skull, cerebro–spinal fluid, gray matter, white matter, and air cavities, respectively. Reprinted (adapted) with permission from Ref. [86]. Copyright ©2021, Academic Press Inc.

Only three electromagnetic theoretical models of electrical stimulation neuromodulation have been established using the above data. The first report is from 2014 when Alvaro Pascal-Leone’s research team at Worcester Polytechnic Institute in the USA built the FEM model of the head through a series of simplifications using the public data of the Visible Human Project (Figure 9) [82,111,114]. This model, through simplification and division, has been used to construct 14 organs including skin, fat, white matter of the brain, and gray matter of the brain. Thus, this model has the largest number of organs for reverse modeling from the existing reports. However, the connection between the eyeball and the brain was broken in the modeling process because of the excessive simplification and combination. Only thin and uneven closed cerebrospinal fluid shells and ventricles were considered in the subarachnoid space, causing difficulties in locating and stimulating special target areas during electrical stimulation and the impact analysis of the correlation between tissues. Second, in 2016, the City University of New York and the Stefan Haufe research team of Columbia University used the Brain VISA software developed based on the US public database [82,111,114] to divide the head data into five parts: skin, skull, cerebrospinal fluid, gray matter, and white matter, and added a cavity through thickening to achieve head modeling (Figure 10) [86]. The team could analyze the effect of electrical stimulation on the brain and the location of EEG source signals using this model. However, because of the oversimplification of this model, its calculation results are not very accurate. The most recent report is from 2019 when Myles McLaughlin’s team at Katholieke Universiteit Leuven simplified the head data into five parts, namely the skin, skull, cerebrospinal fluid, gray matter, and white matter based on the US public database [82,111,114] referencing the Sim4Life data segmentation method developed by Swiss [88,89] to analyze the stimulation effect of AC stimulation on specific areas of the brain (Figure 5) [65]. Effectively analyzing the mechanism of signal propagation in the brain was impossible because the model was too simple. Thus, the authors could not analyze the envelope signals from multichannel signals (The performance analysis of all stimulation models is shown in Table A2).

## 6. The Closed-Loop System and Personalized Digital Twin Brain (Future Work)

### 6.1. The Closed-Loop Neuromodulation System

Most existing electrical stimulation neural regulatory systems are open-loop regulatory systems that require doctors to adjust the stimulation parameters as per the behavior results manually. Its main feature is that the stimulation points are fixed, the stimulation time is determined according to the experience of the medical physicist, and the stimulation effect is not visual, making precise neuromodulation difficult. Particularly in a noninvasive neuromodulation field with combined multi-electrode stimulation, with the increased number of array electrodes, the space of the stimulation parameters expands rapidly, making the identification of the optimal parameters more complicated. The ideal stimulation parameter identification process in an open-loop manner is time-consuming, and the best parameters may not be selected. The closed-loop multi-electrode electrical stimulation neuromodulation system effectively combines the brain’s “time domain” and “space domain” signals [116]. Then, EEG signals are collected while the stimulation is implemented in the time domain. EEG signals are extracted through filters. The changes in the EEG signals with time changes during the stimulation process are analyzed in real time, and the results are parameterized. In the spatial domain, the fMRI images of the brain are collected at a fixed time. In this domain, the brain lesion area is improved relatively slowly over time. Therefore, this domain requires not too rapid brain image acquisition, and the sampling mode can be set according to the characteristics of the disease. For instance, for epilepsy, sampling is performed on the patient upon the attack to analyze the site and the size of the affected area. When stimulation is implemented, sampling is performed once in a magnetic-compatible environment to analyze the focusing status of the stimulation targeting. In continuous treatment, sampling is implemented once every 1–3 weeks to observe the therapeutic effect.

**Figure 11:**
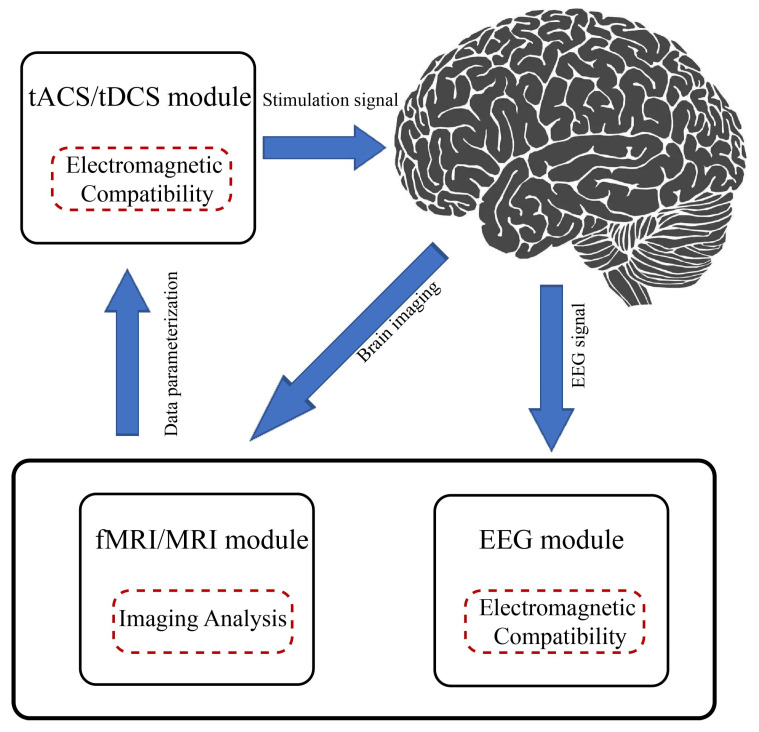
**Architecture of a closed-loop noninvasive electrical stimulation neuromodulation system.** The figure shows a design scheme of a closed-loop noninvasive adaptive neural regulation system, in which the EEG (time domain) and the MRI (space domain) are used to monitor the stimulation effect in real time, and the stimulation parameters are adjusted as per the effect so as to achieve the adaptive control of the system.

Furthermore, the spatial changes in the focus area are analyzed along with the imaging characteristics, and the results are parameterized (Figure 11). The closed-loop system can effectively combine the time-domain result parameters with the space-domain result parameters and adjust the parameter combination (intensity, frequency, and electrode position) of the stimulation system in real time to achieve accurate neuromodulation. In the noninvasive field, closed-loop transcranial direct current stimulation (tDCS) and EEG have been used to explore personalized cognitive training and rehabilitation technology and limb function recovery after stroke [117,118]. The closed-loop neural control system enables the selection of the best control strategy and the simultaneous optimization of the input stimulation and output effect with its adaptive control process and visualization of the control effect.

### 6.2. Personalized Digital-Twin-Brain-Targeted Modeling

**Figure 12:**
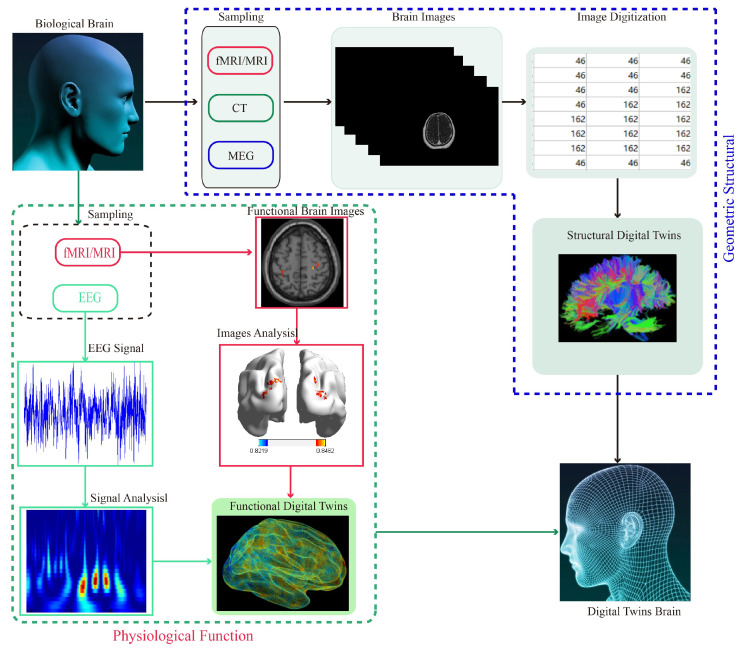
**Personalized digital twin brain targeted modeling.** The figure shows a digital twin brain schematic with electrophysiological characteristics and brain tissue structure characteristics. The head image dataset was obtained using multimodal images; geometric twin construction was achieved by segmentation, reconstruction, and reverse engineering. In the aspect of electrophysiological twinning, the brain network was constructed mainly through collecting and analyzing EEG data, to achieve electrophysiological twinning.

**Figure A2:**
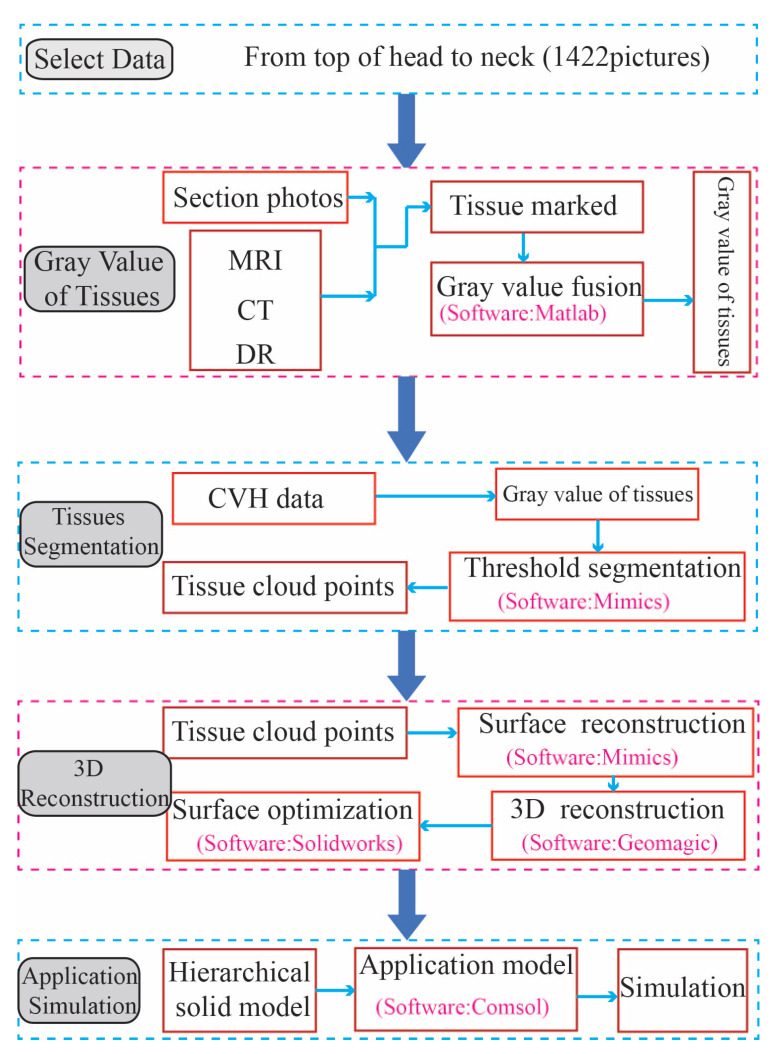
Structural twin digital brain production flowchart.

In terms of targeted stimulation modeling, the existing brain models are all completed using open data sets, leading to the loss of individual differences in the established models; this loss will inevitably affect the targeted stimulation effect of the noninvasive neuromodulation. In particular, noninvasive transcranial electrical stimulation (TES) is more sensitive to tissue characteristics (geometric characteristics and tissue electrical characteristics) [119]. It has a more significant difference in the regulatory effect caused by individual differences. Therefore, a digital twin brain with individual difference characteristics is obtained by indexing the tissue parameters of fresh cadaver multimodal images and fast segmentation of individual (living) head multimodal images through the digital projection method. Additionally, the reconstruction of segmented tissues is becoming a new direction in precise spiritual regulation (Figure 12 and Figure A2). The equipment acquisition and environmental parameters are adjusted to those of the fresh cadaver multimodal image acquisition when collecting the individual head image, and the gray value of the image obtained is consistent, saving time for establishing a personalized digital twin brain.

## 7. Discussion

In this paper, we reviewed the origin and development process of neuromodulation technology based on functional neurological diseases with a focus on the development history of noninvasive neuromodulation technology and typical application cases of modern noninvasive neuromodulation. By analyzing several commonly used invasive neural regulation methods (implantable drug delivery micropump neuromodulation, implantable neural electrical stimulation technology, and deep brain stimulation), it was found that these techniques not only require surgeons with exceptional surgical capabilities but also require an adequate space to fix the invasive device and are constrained by a limited battery capacity. These factors present obstacles to achieving precise neural regulation and burden reduction in patients. According to these cases, the main problems of this technology are as follows: (1) the theoretical models are scarce, and the accuracy needs to be further improved; (2) personalized brain stimulation maps are lacking; and (3) the established model lacks accurate verification methods. Hopefully, more attention will be paid to these problems when developing brain science research, especially regarding personalized treatments and interventions for neurological diseases. The realization of targeted frequency difference signal envelopes by simultaneous use of a multi-electrode combination and loading AC signal with a frequency difference on the electrodes has laid the foundation for noninvasive deep brain electrical stimulation neuromodulation. Furthermore, in the development trend of closed-loop neuromodulation systems, combining the EEG signal in the “time domain” with the fMRI image in the “space domain” has been proposed. The noninvasive deep brain electrical stimulation neuromodulation system can enable the visualization of regulation results and the adaptive adjustment of parameters in the regulation process. A closed-loop system of noninvasive deep brain electrical stimulation neuromodulation was constructed based on this method.

The “digital brain” is the basis for implementing technical theoretical simulation and providing technical guidance for clinical application. This paper summarizes the “digital brain” development process used for the noninvasive electrical stimulation-targeted modeling based on the development of “digital Human” programs in various countries. We conclude that the existing “digital brain” used for noninvasive electrical-stimulation-targeted modeling has the following problems: (1) oversimplification, as it is greatly different from the actual brain structure; (2) excessive use of public data sets and loss of individual differences; and (3) in the living model, the electrical parameters of cadaver tissue are used; thus, the model is inconsistent with the actual situation. This series of problems should drive further research on the “digital brain”. Simultaneously, in the development trend of targeted modeling, we propose to index the tissue parameters of fresh cadaver multimodal images. We suggest using the digital projection method to segment individual (living) head multimodal images rapidly. Then, we suggest reconstructing the segmented tissues to obtain a “digital twin brain” model with personalized tissue structure differences to achieve accurate stimulation guidance for any individual and any target region. In addition, the digital twin brain model we constructed is useful not only in the field of neural regulation but also in the biomechanics of brain trauma [120].

Complex systematic neural engineering is a noninvasive deep brain electrical stimulation neuromodulation system with personalized differences, adaptive adjustment of stimulation parameters, and a visual stimulation effect. This method will be further advanced with the development of medical imaging technology, brain–machine structure technology, and research on brain region function and the brain network. This advancement will be critical for us to understand the brain’s normal and pathological states and implement precise interventions.

Despite the advantages of noninvasive neuromodulation techniques over drug control and invasive methods, significant problems persist in their clinical application. These mainly include the following: (1). The high attenuation of the skull makes it difficult to regulate the intensity of the stimulating signals when they reach the target points, and high-intensity signals can easily burn skin tissue. (2). After the current signal enters the intracranial area, the brain tissue and cerebral effusion have strong signal diffusibility, which poses certain difficulties in focusing the signal [121]. (3). Individual differences make personalized and precise treatment difficult to achieve with a standardized or single model. These issues will limit the clinical application of noninvasive neural regulation and must be urgently addressed.

## 8. Conclusions

This review focused on the treatment of functional neurological diseases. Firstly, the population characteristics of these diseases were reviewed and were found to be large with complex pathology. At present, surgery and drug treatment are still commonly used in clinical practice for treating these diseases. Considering the irreversibility of surgery and the risk of drug resistance caused by long-term use, the medical community has proposed the use of nerve regulation techniques as an alternative treatment.

To gain a more accurate understanding of neural regulation techniques, invasive and noninvasive procedures were separated based on the working mode of the regulation devices. For invasive neural regulation, we focused on analyzing the techniques of implantable drug-delivery micropump neuromodulation, implantable neural electrical stimulation, and deep brain stimulation. Implantable neural regulation is affected by the space for the implanted device, the battery capacity, the high requirements for surgical procedures, and the fixed position of the implantable device that pose certain limitations on precise neural regulation. Therefore, the research hotspot of noninvasive neural regulation techniques involving electrical stimulation is of critical importance and therefore the focus of this review.

Electrical stimulation is divided into tDCS and tACS. Through the literature review, it was found that the main area of action of tDCS is the cerebral cortex; the cortical electrical feedback is used to regulate brain functions, and the signal cannot directly act on the targeted area, which also limits the accuracy of neural regulation. For this reason, we mainly focused on tACS, as it has a strong signal penetration and good operability. According to the literature, tACS has significant advantages, as it is noninvasive deep directional electrical stimulation; however, it has also faced some challenges, the most important of which is the lack of support from high-precision theoretical models. Based on this finding, the research progress in targeted modeling was summarized. We found that establishing digital twin brains based on high-precision structured twinning by using high-precision digital human image data provided theoretical support for tACS research and shows a clear direction for theoretical research in the future.

Based on the findings, we proposed ideas surrounding the precise neural regulation systems and theoretical construction. In terms of the regulation system, a closed-loop system was built to solve the problem of single treatment and overtreatment. The personalized digital twin brain, constructed using individual head image data and electrophysiological data, effectively solved individual differences as well as resolved the theoretical deficiency of precise regulation. The systematic research presented here provides a reference for the future development and research of noninvasive neural electrical stimulation regulation.

## Data Availability

Not applicable.

## Abbreviations and Notes

The following abbreviations and notes are used in this manuscript:

**Abbreviations**	
DBS	Deep Brain Stimulation
tDCS	Transcranial Direct Current Stimulation
tACS	Transcranial Alternating Current Stimulation
TES	Transcranial Electrical Stimulation
TMS	Transcranial Magnetic Stimulation
CSF	Cerebrospinal Fluid
EEG	Electroencephalogram
FEM	Finite Element Method
BEM	Boundary Element Method
MRI	Magnetic Resonance Imaging
**Notes**	
Time domain	This refers to changes in signal over time.
Space domain	This is also known as image space and refers to the expression of
	signals in the image; however, it is not significantly related to time.
Multi-electrode combination	This means that several electrode points are arranged on the cerebral
	cortex, and the combined control over specific areas is realized by
	using the link relationship between electrodes.

## Appendix A

### Appendix A.1. Literature Analysis Flowchart

Shown in Figure A1 below.

### Appendix A.2. Structural Twin Digital Brain Production Flowchart

Shown in Figure A2 below.

### Appendix A.3. Neuromodulation Methods

Shown in Table A1 below.

**Table A1:** Neuromodulation methods.

Types	Methods	Location	Advantage	Disadvantage
Invasive	DDM [29,30,31]	Subarachnoid space	Accurate drugs	Resistant to drugs
	NES [17,18,19,20,21,22]	Brain, spinal cord, peripheral nerves, nerve plexus, autonomic nerves	Accurate stimulation	Operative difficulty and implantation space are higher
	DBS [17,18,19,20,21,22,23,24,25,29,30,31,32,33,34]	Brain	Accurate stimulation	Operative difficulty and implantation space are higher
Non-Invasive	TMS [76,77]	Outside surface of the brain	Deep brain, High precision,	Organic changes, Large attenuation
	tDCS [23,44,45,70,71,76]	Cerebral cortex	Easy operation	Small depth (only in the cortex)
	tACS [24,73]	Cerebral cortex	Deep brain, Easy operation, High precision	Difficult to accomplish, theoretical support is lacking

DDM: Implantable drug-delivery micropump, NES: Implantable neural electrical stimulation.

### Appendix A.4. Stimulation models

Shown in Table A2 below.

**Table A2:** Stimulation models.

Models	Tissues	Advantage	Disadvantage
Single-layer head model [77]	None	Simple, high speed of calculation	Large error and no practical significance
Multilayer sphere model [78,79]	Cerebral white matter	Simple, high speed of calculation	Large error and no practical significance
Integrated Digital Human model (Sim4Life) [88,89]	Cortex, muscles, skeletons, etc.	Approximate to the real human body	Computationally complex, highly resource-consuming, not available for secondary development, incapable of conducting electrical stimulation simulation
Simplified Digital Human model [82,83,84,85,93,94,95,96,97,98,99,100,101,102,103,104,105,106,107,108,109,110,111,112,113,114]	No nerves, soft tissues, or other small tissues	Reduces the difficulty of modeling and the capability of conducting electrical stimulation simulation	Discards soft tissue and cannot fully express human tissue
Chinese Digital Human model [76,87,90,91,92,115]	A total of 110 types of tissues, including cortex, muscles, and skeletons	Consistent with the real human body, capable of conducting electrical stimulation simulation	Difficult to model, computationally complex, highly resource-consuming
